# Effects of Multivitamins and Known Teratogens on Chick Cardiomyocytes Micromass Culture Assay

**Published:** 2013-09

**Authors:** Samreen Memon, Margaret Pratten

**Affiliations:** 1 ** Department of Anatomy Liaquat University of Medical Sciences, Pakistan**; 2 School of Biomedical Sciences, University of Nottingham, UK

**Keywords:** Chick cardiomyocyte, Environmental teratogens, Micromass culture, Multivitamins

## Abstract

***Objective(s):*** This study aimed to find out whether the chick cardiomyocyte micromass (MM) system could be employed to predict the teratogenecity of common environmental factors. Different multivitamins and over the counter drugs were used in this study.

***Materials and Methods:*** White Leghorn 5-day-old embryo hearts were dissected and trypsinized to produce a cardiomyocyte cell suspension in Dulbecco's Modified Eagle's Medium. The cultures were incubated at 37^0^C in 5% CO_2_ in air, and observations were made at 24, 48 and 144 hr, for the detection of cell beating. Cellular viability was assessed using the resazurin assay and cell protein content was assessed by the kenacid blue assay. It was observed that while not affecting total cell number folic acid, vitamin C, sodium fluoride and ginseng did not significantly reduced cell activity and beating. However cadmium chloride significantly reduced the beating, cell viability and cell protein content in micromass cultures.

***Results:*** The results demonstrate the potential of the chick cardiomyocyte MM culture assay to identify teratogens/embryotoxins that alter morphology and function, which may result in either teratogenic outcome or cytotoxicity.

***Conclusion: ***This could form part of a screen for developmental toxicity related to cardiac function.

## Introduction

Teratogenecity testing of different toxins, chemicals and drugs, which pregnant mothers come across in everyday life, is essential. Screening of new chemicals with potential toxicity would allow medical experts to help pregnant women in avoiding direct contact with these potentially hazardous substances. Currently most teratogenecity assays are utilized *in vivo *animal studies to attain the goal of detecting chemical hazards (1). In recent years, scientists started using *in vitro *methods to overcome the intrinsic problems and differences in animal teratology studies. These methods are now well established and invaluable for conducting these studies, and are very useful for the screening of chemicals (2). The *in vitro* tests are less expensive, quicker, and much more reproducible. There is now an absolute need for alternatives to conventional animal-based methods due to the fact that every year hundreds of drugs are introduced to the market and pregnant women are exposed to thousands of toxic substances in everyday life (3). The European Union (EU) White Paper published in 2001 suggested the organization of testing requirements for approximately 30,000 chemicals marketed before September 1981 (Registration, Evaluation and Authorization of Chemicals, ‘REACH’) (4). There are many available *in vitro* tests, e.g. hydra regeneration assay, the frog embryo teratogenesis assay (FETAX), drosophila assay, which detect the developmental and reproductive toxicity in mammals and other primates. Out of all the *in vitro* tests, three are validated as embryo toxicity assays by the ECVAM advisory committee (ESAC). These are the embryonic stem cell test, the micromass (MM) test and the whole embryo culture test (-).

The MM system involves the culture of primary cells, isolated either from the mesencephalon, heart or the limb buds of developing embryos, plated at high density (8). The basic principle of assay is the potential of teratogens to disrupt normal differentiation of primary embryonic cells in vitro. Several species have been used for micromass cultures: rat mouse (-); and chick (-). The chick MM assay utilizes primary cells obtained from midbrain, limbs or heart of chick embryos. Once the cells are cultured in high density they can then be exposed to the test chemicals in replicates and observed for cytotoxic effects by the use of relevant endpoints, i.e. differentiation, and cytotoxicity assays (19).

**Table 1:** Morphological scoring system to determine contractile activity of cardiomyocytes

Numerical morphological score	Contractile activity
0	No contractile activity
1	Few contracting foci
2	Numerous contracting foci
3	Entire plate contracting

Folic acid and vitamin C are water soluble vitamins. An adequate supply of dietary folate and vitamin C in pregnancy is proved to be essential for normal embryonic development (-). Cadmium chloride is a non essential heavy metal with no known biological role in humans. Cadmium exposure gives rise to many developmental defects in chick including limb and anterior body wall defects (23, 24). Different forms of fluoride, including sodium fluoride have been added to drinking water by many countries to protect against dental caries. Although it is an essential component for humans at low concentrations but if consumed more than 1ppm, it causes fluorosis (25). Ginseng, an herbal medicine, has long been used as a tonic for prolonging life span, and is available without prescription. It is employed by different people, including pregnant women (26). This study aims to evaluate the effectiveness of micromass culture system as screening method for in* vitro* toxicity assay, using different vitamins and known environmental toxins. 

## Materials and Methods

***Chemicals and solutions***

Folic acid, vitamin C (ascorbic acid), sodium fluoride, cadmium chloride and ginseng (Ginsengoside 1), Kenacid Blue (KB), Hank’s balanced salt solution (HBSS), trypsin-EDTA, horse serum, penicillin/streptomycin, resazurin, resorufin were purchased from Sigma-Aldrich (Poole, UK). Dulbecco’s Modified Eagles Medium (DMEM) and L-glutamine were purchased from Cambrex Bio Sciences Wokingham, UK, Ltd. Fetal calf serum was purchased from Autogen Bioclear (Wiltshire, UK). The test chemicals were added within 30 min of being prepared, and applied 24 hr after the cultures were seeded.

***Micromass culture preparation***

White Leghorn chicken eggs (Henry Stewart Co., Louth, UK) were incubated on an automatic egg rotator in an incubator at 37°C and 100% humidity for 5 days. The organs at day 5 have still the ability to differentiate and can be clearly identified (13, 15). The embryos were removed from the vascular network with bent forceps, and the hearts were dissected out and pooled by placing in 5 ml of sterile 50% (v/v) horse serum in HBSS on ice. Once the hearts from all the embryos were dissected, they were dissociated with 4 ml 1% trypsin/EDTA at 37°C for 20 min agitated at every 5 min. The culture medium (Dulbecco’s modified eagles medium (DMEM) supplemented with 10% fetal calf serum, 200 mM L-glutamine and 50 U/ml penicillin/50 µg/ml streptomycin) was added to neutralise the activity of the trypsin, and centrifuged at RT at 1500 rpm for 5 min. The pellet was resuspended in 1 ml culture medium and 20 µl of the cell suspension (3x106cells/ml) was plated into each well of 24-well tissue culture plate. The cells were allowed to attach for 2 hr at 37°C and 5% (v/v) CO2 in air, before addition of 500 µl culture medium. After 24 hr, 500 µl of culture medium containing either the diluted chemical or culture medium alone was added. Once the cells are cultured in high density they can then be exposed to the test chemicals in replicates and observed for cytotoxic effects by the use of relevant endpoints, i.e. differentiation, and cytotoxicity assays (19).

*End points*

The resazurin reduction assay, kenacid blue assay and differentiation assay were performed. 

***Cellular differentiation***

The cultures were inspected morphologically for cardiomyocyte contractile activity at 24, 48 and 144 hr under light microscope and observations were made according to the scoring method shown in Table 1. 

*Resazurin reduction assay*

The resazurin assay was performed on day 6 following explantation (27). The medium was removed from the 24 well plates and replaced with 500 µl resazurin solution. The plates were then incubated for one hour at 37^o^C and 5% (v/v) CO_2_ in air. The optical density was read using a FLUOR star plate reader, excitation wavelength of -530±12.5 nm, with a gain of 10. 

***Kenacid blue total protein assay***

The same cells subjected to the resazurin reduction assay were assayed for total protein using the kenacid blue assay. Wells were aspirated and 300 µl KB fixatives was added and allowed to evaporate overnight at 4^o^C. KB working solution (400 µl) (28) was added to each well and the plate placed on a plate shaker for at least 2 hr. Excess stain was removed and cells were quickly rinsed before being washed in 400 µl of washing solution for 20 min with agitation. The washing solution was replaced with 400 µl of desorb and gently agitated on the plate shaker for one hr. The optical density was read on an ASYS HITEC Expert 96 plate reader with a reference filter of 405 nm, and a reading filter of 570 nm. 

*Statistical analysis*

Statistical analysis was performed using Prism 5 (Graph pad Software Inc. San Diego, USA) for three independent runs. All results with different dose concentrations were compared using one way ANOVA with Dunnet’s multiple comparison *post hoc* test, with *P*< 0.05 was considered statistically significant.

## Results

*Folic acid*

**Figure 1:**
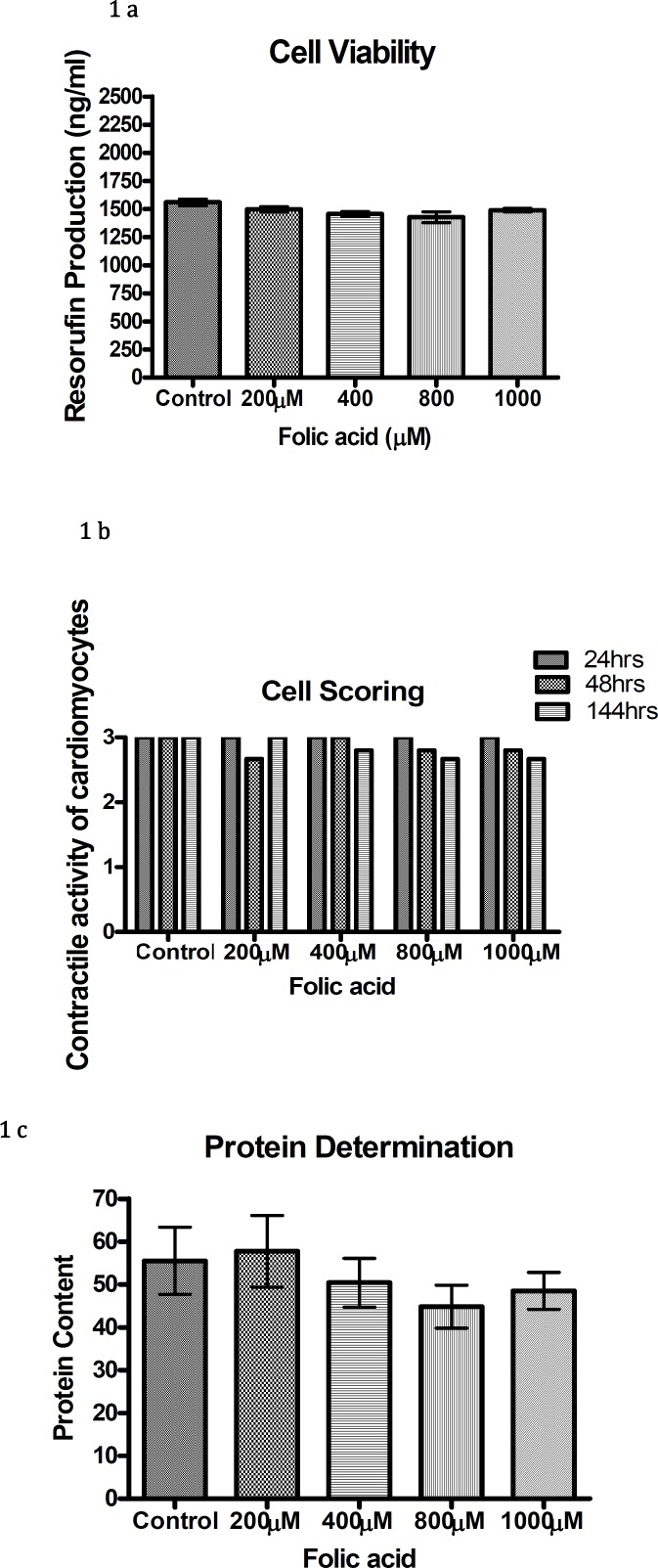
Resorufin production (a), contractile activity of cardiomyocytes (b) and protein content (c) with different concentrations of folic acid

The graphs for resorufin production, cellular beating and protein content of folic acid (200 µM-1 mM) showed no significant difference to the controls (Figure 1a, 1b and 1c). 

***Vitamin C***

**Figure 2:**
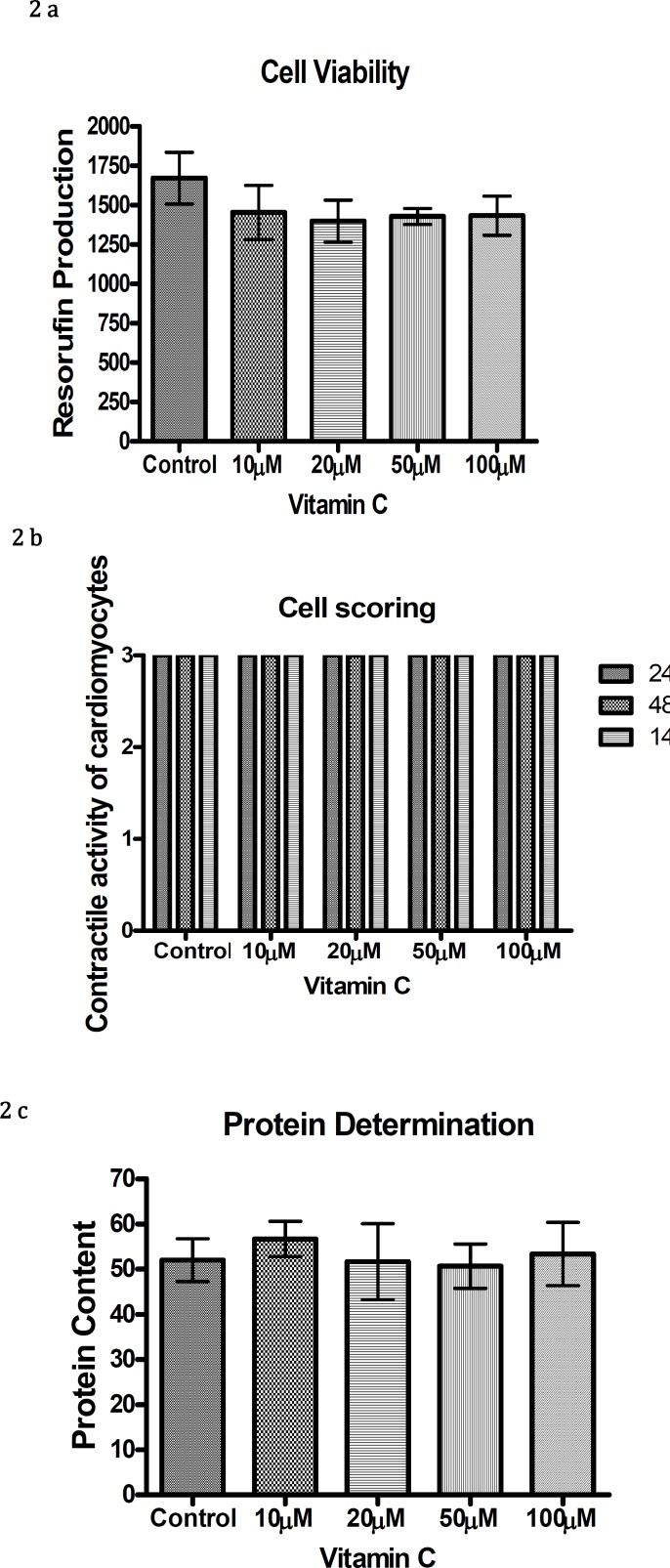
Resorufin production (a), contractile activity of cardiomyocytes (b) and protein content (c) with different concentrations of vitamin C

Exposure of chick cardiomyocytes to different concentrations of vitamin C (10 µM-100 µM), over a period of 144 hr did not show any reduction in cell viability, cellular differentiation, cellular protein level as shown in Figure 2a, 2b, 2c respectively.

*Cadmium Chloride*

**Figure 3:**
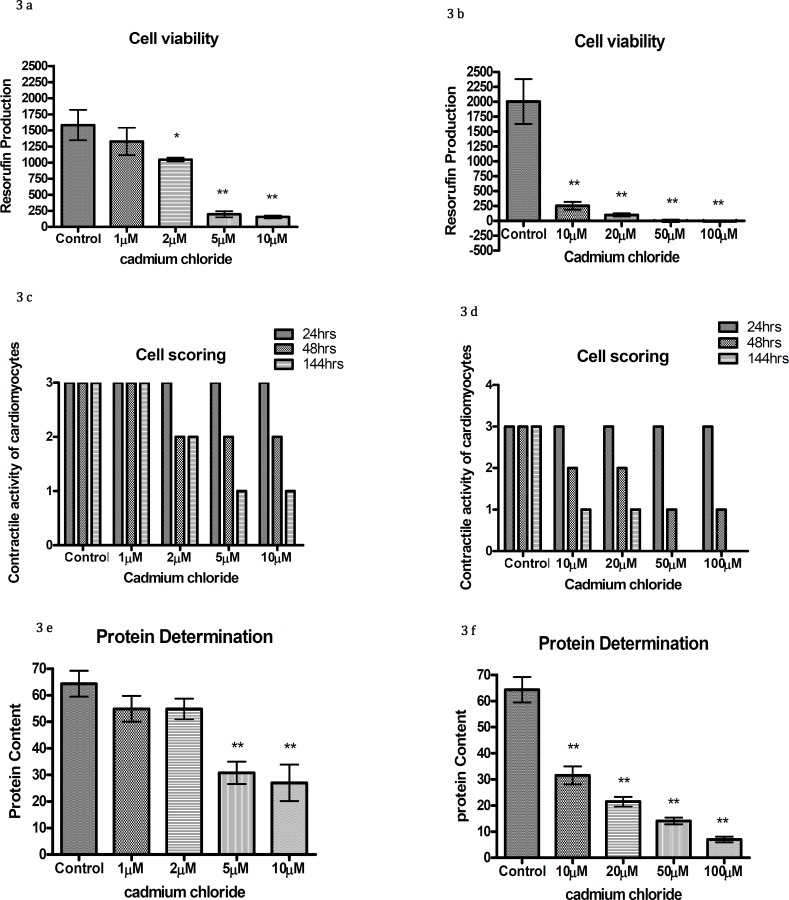
Resorufin production (a & b), contractile activity of cardiomyocytes (c & d) and protein content (e & f) with different concentrations of cadmium chloride

The cardiomyocytes treated with various concentrations of cadmium chloride (1 µM to 100 µM) showed that the cultures exposed to 1 µM or more had a significantly decreased contractile activity (3 c,d), while resorufin production and protein content was significantly different to controls from 5µM and above (Figure 3 a, b, e, f). 

*Sodium fluoride*

**Figure 4:**
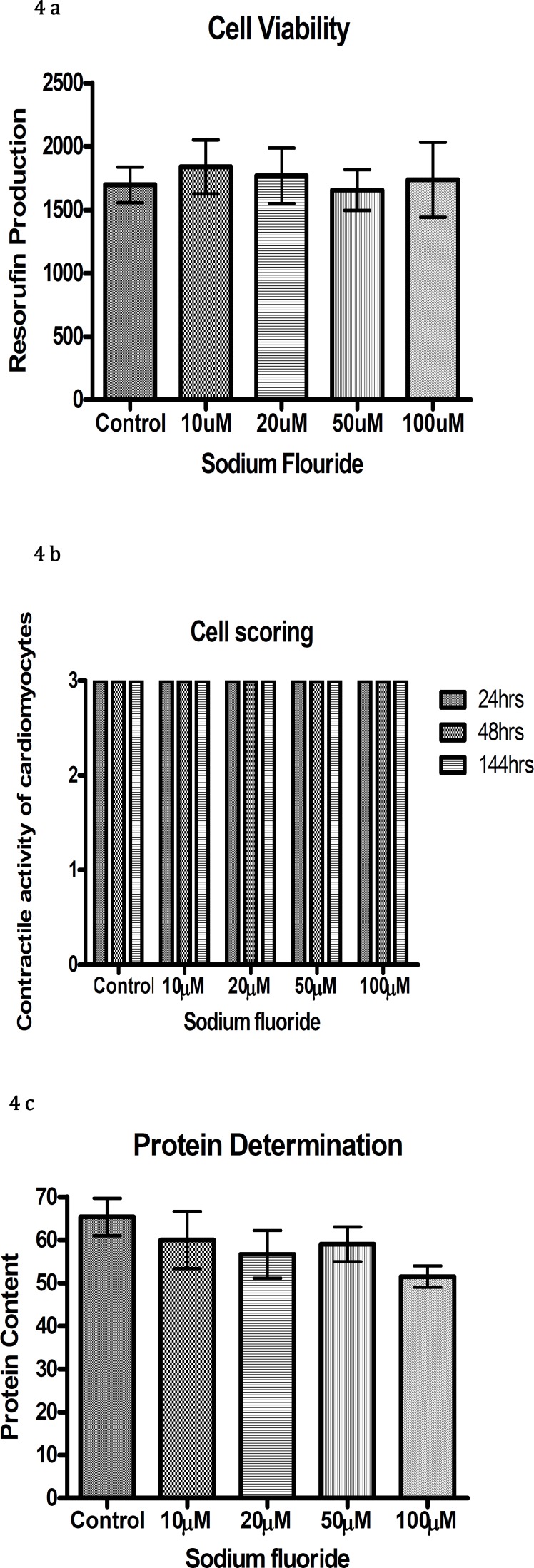
Resorufin production (a), contractile activity of cardiomyocytes (b), and protein content (c) with different concentrations of sodium fluoride

Exposure of chick cardiomyocytes to 10µM to 100µM of sodium fluoride over a period of 144 hr did not cause a reduction in resorufin production, cellular differentiation or protein content (Figure 4 a,b, c).

*Ginseng*

**Figure 5:**
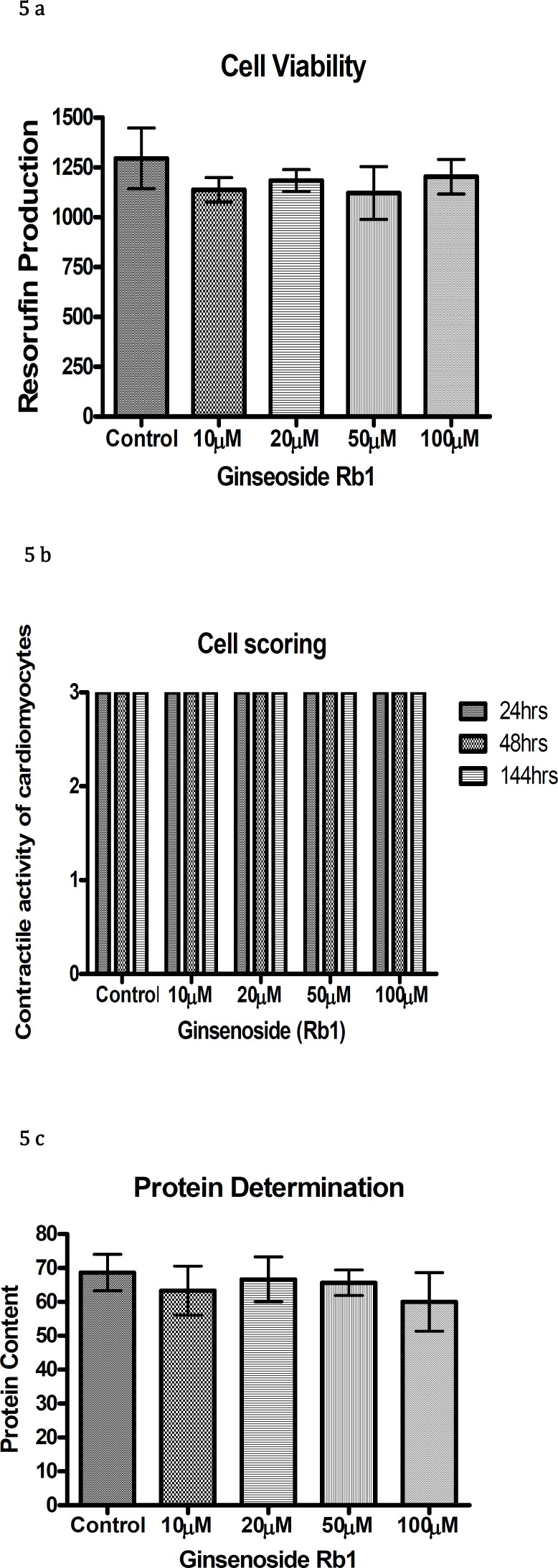
Resorufin production (a), contractile activity of cardiomyocytes (b), and protein content (c) with different concentrations of ginsenoside (Rb1)

Ginsenosides (Rb1) exposure to chick cardiomyocytes in the range between 10 µM to 100 µM showed no reduction in resorufin production, cellular differentiation or protein content during the entire duration of culture as shown in Figure 5 a,b,c. 

## Discussion

Chicks are very appealing species for teratogen screening because of their ease of storage during embryonic development, the avoidance of the sacrifice of the mother and their fast development. Brown *et al* (29) reported that no significant differences could be observed when comparing rat and chick limb bud MM responses with a range of potential teratogens. In this study the reliability of chick cardiomyocyte micromass culture was tested with known non-teratogens and few know teratogens. The cardiomyocytes were treated with different doses of folic acid ranging from 1 µM to 1 mM. It was observed that even at 1mM folic acid did not show any toxic effects on chick cardiomyocytes, which is consistent with the amount of folinic acid used to treat the malformations of branchial arch derived structures in rats in whole embryo culture by (30). Recently studies conducted on animals as well as on humans suggest that folic acid might also be useful in decreasing other birth defects; in particular congenital heart defects (-). Cadmium chloride is generated as a result of waste disposal, coal combustion and phosphate fertilizers manufactures. It is used in this study in a range of 1 µM to 100 µM, which is in line with other cell culture studies, which used cadmium in micro molar concentrations (34, 35). The chick exposed cardiomyocytes stopped beating and cell viability assays showed a reduction in viability and total proteins. In studies conducted by Chow and Cheng (36) and Chen *et al* on zebra fish embryos at early developmental stages, showed that cadmium had toxic effects on early development and when these embryos were exposed to higher doses of cadmium, they showed developmental defects in the head and neck region, heart malformations, and had altered axial curvatures (37). Fluoride is a naturally occurring component of water and high levels of it are usually found in mountainous areas (38). 

Sodium fluoride was used in a range of 10 µM-100 µM to detect any potential teratogenic effect on chick embryonic hearts using micromass culture. It was observed that, the concentrations used in this study had no toxic effect on the developing chick heart. Verma and Sherlin showed skeletal abnormalities with some subcutaneous haemorrhages (39). In another study no embryonic defects were seen when female rats and rabbits were given 27 mg/kg/day sodium fluoride in drinking water. Effects were observed at higher concentrations due to the reason that concentrations higher than that dose were unpalatable and pregnant rats and rabbits were reluctant to take any food even beyond that dose (40). Our results are consistent with these results as low doses of sodium fluoride used in this study also showed no developmental defects. An *in vitro* study on frog embryos showed developmental abnormalities in frog embryos at higher doses but not at lower doses. Ginseng is one of the most well-known natural herbal medicines, used widely in the treatment of various diseases (41, 42). No significant teratogenic effects were observed at the doses (10 µM-100 µM) used in this study. However previous work with mouse and rat embryos cultured *in vitro*, showed that ginsenoside when used in the range of 10-50 µg/ml, had toxic effects on developing embryos (43, 44). However the two species appeared to have toxic effects at different concentrations of ginsenosides. The rat embryos proved to be more sensitive than mouse embryos (45). The inconsistency between our results and previous results on rat and mouse embryos could be due to the dose of ginsenoside used in this study or due to the species difference. The mechanism of ginseng toxicity is still unclear but it might be due to alterations in calcium channels (46). 

## Conclusion

It is concluded from this study and previous work done on chick heart micromass culture is a reliable assay and might be used as an alternative for *in vitro* toxicity assays.
